# Antibacterial and Antibiofilm Effect of Honey in the Prevention of Dental Caries: A Recent Perspective

**DOI:** 10.3390/foods11172670

**Published:** 2022-09-02

**Authors:** Juraj Deglovic, Nora Majtanova, Juraj Majtan

**Affiliations:** 1 Department of Dental Medicine, Faculty of Medicine, Slovak Medical University, Limbova 12, 833 03 Bratislava, Slovakia; 2 Department of Ophthalmology, Faculty of Medicine, Slovak Medical University, Limbova 12, 833 03 Bratislava, Slovakia; 3 Laboratory of Apidology and Apitherapy, Department of Microbial Genetics, Institute of Molecular Biology, Slovak Academy of Sciences, Dubravska Cesta 21, 845 51 Bratislava, Slovakia; 4 Department of Microbiology, Faculty of Medicine, Slovak Medical University, Limbova 12, 833 03 Bratislava, Slovakia

**Keywords:** honey, antibacterial, quality, dental medicine, *S. mutans*

## Abstract

The successful application of honey in wound care management has been achieved due to honey’s potent antibacterial effects, characterised by its multifactorial action. Impressive clinical efficacy has ignited its further use in diverse clinical disciplines, including stomatology. Indeed, there is increasing usage of honey in dental medicine as a preventive or therapeutic remedy for some periodontal diseases mainly associated with bacteria, such as dental caries, gingivitis and mucositides. Dental caries is undoubtedly a major oral health problem worldwide, with an increasing tendency of incidence. The purpose of this perspective review is to describe the recent progress in the laboratory and clinical use of honey in the prevention of dental caries, with emphasis on the antibacterial and antibiofilm effects of honey. The role of honey in the cariogenic process is also discussed. In addition, the quality of honey and the urgent in vitro evaluation of its antibacterial/antibiofilm properties before clinical use are highlighted. Findings based on data extracted from laboratory studies demonstrate the pronounced antibacterial effect of different honeys against a number of periodontal pathogens, including *Streptococcus mutans*. Although the promising antibiofilm effects of honey have been reported mainly against *S. mutans*, these results are limited to very few studies. From a clinical point of view, honey significantly reduces dental plaque; however, it is not superior to the conventional agent. Despite the positive in vitro results, the clinical effectiveness of honey in the prevention of dental caries remains inconclusive since further robust clinical studies are needed.

## 1. Introduction

Dental caries is considered to be a global health priority in the treatment of oral diseases. According to the Global Burden of Disease 2015 study, 3.5 billion people worldwide had dental conditions, predominantly untreated dental caries [1]. Overall, the global burden of untreated dental caries for primary and permanent dentition has remained relatively unchanged over the past 30 years [2]. Dental caries is the localised destruction of susceptible dental hard tissues (enamel and dentine) by weak organic acidic by-products from the bacterial fermentation of dietary carbohydrates (sucrose) [3]. It is a multifactorial aetiology disease where bacterial species (mainly streptococci, lactobacilli and bifidobacteria) are organised in sessile communities, supragingival dental plaque, which is affected by salivary flow and composition, the consumption of dietary sugars and preventive behaviours. The oral cavity may harbour over 700 prokaryotic species, including bacteria associated with periodontal diseases and those that possess health-promoting properties [4]. These commensal bacteria are able to buffer acidic pH, reduce gingival inflammation or inhibit the growth of pathogens.

The sugar-fermenting, acidogenic species *Streptococcus mutans* is the main causative agent of dental caries; however, DNA- and RNA-sequencing studies of carious lesions have revealed a consortium of multiple microorganisms [5]. The oral microbiome of healthy subjects differs from the human microbiomes of supra- and subgingival dental plaque, where 13 genera, highly abundant with high prevalence, have been revealed, including *Streptococcus*, *Corynebacterium* and *Rothia* [6]. DNA studies have allowed the identification of the microbial cells within dental plaque biofilms where extracellular matrix substances, such as carbohydrates, nucleic acids, proteins and lipids, are critical components that maintain the spatial arrangement of cells and coordinate cellular functions throughout the matrix.

Dental caries is a classic biofilm-induced disease that causes the destruction of mineralised tooth tissue. Cariogenic bacteria are required but not sufficient to cause dental caries because the formation of cariogenic biofilms is dependent on the host diet [7]. A sugar-rich diet promotes the assembly of extracellular matrix polymeric substances and enhances the accumulation of acidogenic and acid-tolerant microbiota. An accumulation of bacteria producing organic acids results in a local decrease of pH value and, thus, causes the demineralisation of the dental hard tissue. The demineralisation process starts with damage to enamel and dentine, but this process can be reversed by the uptake of calcium, phosphate and fluoride [8]. Repeated demineralisation over a prolonged period leads to the formation of dental caries.

## 2. Strategies for Preventing Dental Caries

Dental caries is undoubtedly a major oral health problem. A very recent epidemiological study showed that the global number of cases of caries of permanent teeth increased by 46.1% (95% uncertainty interval, 42.0% to 50.3%) from 1990 to 2019 [9]. The fast increase in the dental caries burden suggests the need to re-evaluate the current insurance scheme and strengthen efforts to prevent caries. 

It has recently been suggested that dental caries prevention and management is about controlling risk factors to maintain a balanced intraoral biofilm ecology that guards against a continuing low pH [10]. Based on current knowledge, the authors classified dental caries as a non-communicable disease. According to a World Health Organization oral health report published in 2022, minimally invasive intervention approaches to prevent and treat caries should be applied to extend the longevity of natural teeth and to avoid unnecessary pain, infection and permanent damage to teeth [11]. Among the essential components for the preventive management of dental caries, dental plaque control is one of the key approaches to reducing the development of dental caries. Tooth brushing for mechanical plaque control and interdental cleaning tools are the most common oral hygiene practices. Using floss or interdental brushes in addition to toothbrushing may reduce plaque more than toothbrushing alone [12]. In addition, therapeutic types of mouthwash represent another adjunctive tool, along with a regular oral hygiene routine. The reason for using mouthwash is to prevent biofilm accumulation rather than eradication. Analysis of the current state of the evidence on the efficacy of mouthwashes in terms of dental plaque reduction has been conducted based on systematic reviews published between 2012 and 2017 [13]. All analysed systematic reviews are in complete agreement that mouthwashes based on chlorhexidine and essential oils provide statistically significant improvements in terms of plaque prevention.

The frequent use of chlorhexidine gluconate and other chemical antiseptics raises concern regarding the development of acquired bacterial resistance. A wide range of pathogenic bacteria, including nosocomial pathogens, has developed reduced susceptibilities to chlorhexidine gluconate [14]. Furthermore, long-term use of chlorhexidine may be characterised by specific unwanted adverse effects such as parotid gland swelling, pigmentation of soft tissue and teeth, taste alteration and a burning sensation [15].

Nowadays, a variety of natural products or their active ingredients, such as curcumin, honey, green tea extract and aloe vera, have become a part of dental treatment due to their reduced toxicity, wide availability and cost-effectiveness. Plant- and insect-derived natural products offer a multifactorial mode of antibacterial action and, therefore, represent an attractive active substance in mouthwashes. In fact, many natural products have already been clinically tested in the prevention of dental plaque and compared to conventional therapy. A very recent systematic review and meta-analysis evaluated the efficacy of curcumin mouthwashes in controlling dental plaque [16]. The comparable efficacy of curcumin and chlorhexidine in reducing dental plaque was revealed.

## 3. Honey—Its Composition and Antibacterial/Antibiofilm Properties

Honey is the natural sweet substance produced by honey bees from the nectar of plants, secretions of the living parts of plants or excretions of plant-sucking insects [17]. Legislation sets the limits for parameters that define the physicochemical, organoleptic and microscopic characteristics of honey. However, the complexity of honey does not allow the exact limits for legislative criteria to be defined. Similarly, variation in composition leads to variations in biological activity, including antibacterial and antioxidant activity.

Honey is a super-saturated solution of sugars (up to 80% of the product’s total composition) enriched with other minor components, including amino acids, peptides, proteins/enzymes, acids, lactones, minerals and polyphenols [18]. However, these components, found at low concentrations in honey, are necessary to determine its taste, colour and aroma, in addition to the wide spectrum of health-promoting and therapeutic properties. The detailed composition of honey has already been reviewed elsewhere [18,19,20].

### 3.1. Antibacterial Effect of Honey against Oral Pathogens

Honey’s antibacterial activity has been considered one of the most important biological properties that determine honey as a functional food [21]. Therefore, a plethora of studies have characterised the antibacterial effect of honey of different botanical and geographical origins. A low pH and water activity and a high sugar content (osmolarity), hydrogen peroxide (H_2_O_2_), gluconic acid and antimicrobial proteins/peptides have been identified as the major factors responsible for the antibacterial effects of honey [22] (Table 1). All these factors are present in every type of honey regardless of its botanical and geographical origin; however, their concentrations can vary from honey to honey. Some of these antibacterial compounds, such as bee-derived defensin-1 and major royal jelly protein 1 (MRPJ1), have also been suggested as qualitative parameters of honey authenticity [23,24,25]. The antibacterial activity of honey can be enhanced by phytochemicals such as methylglyoxal (MGO) and polyphenols, including flavonoids that are present in certain types of honey (e.g., manuka honey) [26,27,28].

Recent studies have provided compelling evidence that H_2_O_2_ plays a key role in antibacterial activity [29]. It is enzymatically produced by glucose oxidase (GOX), a honeybee enzyme that is activated upon dilution of honey. In most cases, the maximum H_2_O_2_ concentration is reached at honey dilutions ranging from 15% to 50% (w/v). Dark-coloured honeys, such as buckwheat and heather honeys, often produce higher amounts of H_2_O_2_ than light-coloured honeys [29]. 

In addition to the enzymatic generation of H_2_O_2_, this compound can be produced as a result of the pro-oxidant activity of polyphenols. Thus, polyphenols, often reported in honey samples, may contribute to or modulate antibacterial activity. In the presence of transition metal ions (Cu and Fe) and peroxides, polyphenols can act as pro-oxidants by accelerating hydroxyl radical formation and oxidative strand breakage in DNA [30]. In fact, polyphenols work in two ways to promote antibacterial activity: by directly producing H_2_O_2_ and by reducing Fe (III) to Fe (II), which triggers the Fenton reaction to create more potent reactive oxygen species such as hydroxyl radicals. A key factor in determining whether polyphenolic compounds exhibit antioxidative or antibacterial properties is pH value [31].

The oxidative process, resulting in hydroxyl radical formation, can kill cells if their accumulation is not controlled since hydroxyl radicals break nucleic acids, carbonylate proteins and peptides and peroxidate lipids of bacterial cell membranes [29]. 

Due to the conventional usage of honey in wound care, most studies characterising the antibacterial activity of honey have focused on wound bacterial pathogens, including *Staphylococcus aureus* and *Pseudomonas aeruginosa*. However, recent progress in the clinical application of honey opens new avenues for the antimicrobial usage of honey in the treatment of periodontal diseases [46]. In fact, Hbibi and co-workers [46] conducted a systematic review in order to evaluate the scientific evidence regarding the antimicrobial efficacy of honey against periodontopathogens. Based on the 16 papers selected and analysed, including five clinical studies, the authors concluded that there is a positive in vitro antimicrobial effect of honey when used either undiluted or diluted on periodontopathogens such as *Aggregatibacter actinomycetemcomitans*, *Porphyromonas gingivalis*, *Streptococcus gordonii*, *Campylobacter* spp., *Campylobacter rectus* and *Eubacterium nodatum*. On the other hand, the reported data are rather heterogenous, and major differences were found in honey botanical origin, honey dilution forms (w/v, v/v, w/w), bacterial growth conditions and the origin of tested bacteria (reference strain vs. clinical isolate). Manuka honey is the most commonly studied. It has been shown to be active against a variety of bacteria, including dental plaque-associated bacteria, both as planktonic and biofilm organisms [47,48,49,50]. Gram-negative periodontal pathogens such as *P. gingivalis*, *Prevotella intermedia* and *Fusobacterium nucleatum* are more sensitive to manuka and clover honey than the Gram-positive streptococci [49]. Furthermore, *Streptococcus mutans* is more resistant to honey types, with minimal bactericidal concentrations being in the range of 25% to 50% (w/v). In another study, non-manuka honey inhibited the bacterial growth of *S. mutans* at concentrations of between 12.5% and 25% [51]. On the other hand, no inhibition of bacterial growth of *S. mutans* was documented at non-manuka honey concentrations of 5%, 10%, 20% and 40% (v/v) [52]. *S. mutans*, a notably acidic-tolerant bacterium, is the most common bacterium associated with dental caries. As mentioned above, honey is able to inhibit the bacterial growth of *S. mutans* only at very high concentrations, whereas low concentrations are needed to inhibit oral commensals [50]. MGO and H_2_O_2_, major antibacterial compounds of manuka and non-manuka honey, respectively, act differentially against oral bacteria. MGO has been shown to be active against oral pathogens and commensals at a concentration of less than 0.31%. On the contrary, H_2_O_2_ did not inhibit bacterial growth up to the tested concentration of 2 mM [50]. The level of H_2_O_2_ accumulated in diluted honey varies significantly. Diluted blossom honey and honeydew honey generate H_2_O_2_ at concentrations ranging from 32 to 3376 µM and 300 to 3400 µM, respectively [28,41]. According to average H_2_O_2_ values, honeydew honey (1.8 mM) is most potent in comparison to blossom honey (0.7 mM) [28,41].

Although the studies performed have suggested the limited efficacy of honey against *S. mutans*, a very recent laboratory observation with different Greek honey types and manuka honey revealed that all tested honey samples were highly effective against *S. mutans* [53]. The average MIC values of citrus (n = 20), *Satureja* spp. (n = 20) and oregano and sage (n = 20) honey samples against *S. mutans* were 6.2%, 4.5% and 6.25% (w/v), respectively. The antibacterial effect is significantly reduced in citrus honey after the decomposition of H_2_O_2_ by the enzyme catalase; however, it remains stable in *Satureja* spp. and oregano and sage honeys. Furthermore, if artificial saliva is used as a honey diluent, the antibacterial activity is even slightly enhanced.

Apart from H_2_O_2_ and MGO, the recently explored exosome-like extracellular vesicles in honey [54] display a pronounced antibacterial effect against *S. mutans,* mediated through nanomechanical alterations resulting in membrane damage [55]. Detailed molecular characterisation of these honey-derived vesicles has further revealed that MRJP-1, defensin-1 and jellein-3 are cargo proteins in vesicles. All these three bee-derived proteinous compounds exert antibacterial activity against Gram-positive bacteria, and two of them, namely, MRJP1 and defensin-1, also do so against Gram-negative bacteria [23,32,33,36,56]. 

One of the options for increasing the antibacterial effect of honey against *S. mutans* is the supplementation of honey with other ingredients through synergistic antibacterial activity. 

Cranberry extracts, when paired with manuka honey, show stronger antibacterial action compared to the individual extract or honey. The antibacterial activity of this combination has been compared with commercial mouthwashes in a well-diffusion assay, where the mixture of honey and cranberry extract showed a significantly larger inhibition zone than mouthwashes [57]. Similarly, acacia honey, myrtle and pomegranate extract were able to inhibit the cariogenic bacterium *S. mutans* via synergistic effects [58].

### 3.2. Antibiofilm Effect of Honey against Oral Pathogens

Dental biofilms play a vital role in caries initiation and development. Therefore, the prevention and/or eradication of dental plaque biofilm is an important approach in the management of dental caries. There are several strategies for inhibiting the formation of cariogenic bacterial biofilms as well as for disrupting already-formed biofilm [59]. From practical and economic points of view, the prevention of plaque biofilm formation is a key step in maintaining good oral health. 

Clinical outcomes from honey-treated wounds associated with biofilm suggest that honey is an effective antibiofilm agent with anti-adherent and biofilm-dispersing properties [60]. Thus, the vast majority of in vitro, in vivo and clinical studies using honey as an antibiofilm product have been carried out against bacterial wound pathogens, particularly *Staphylococcus aureus* and *Pseudomonas aeruginosa*. Furthermore, several antibiofilm compounds found in honey have been characterised in mono- and multispecies biofilms. Taking into account the composition of honey, the most promising group of compounds possessing antibiofilm activity are antimicrobial peptides—defensins. In fact, insect defensins are potent antibacterial and antibiofilm peptides [61]. Defensin-1, a regular but variable-concentration peptide found in every type of honey, has been found to be involved in honey’s antibiofilm activity [62]. Furthermore, a recombinant form of bee-derived defensin-1 successfully reduces the viability of *S. aureus* and *P. aeruginosa* cells within established polymicrobial biofilms [63]. It also significantly affects the biofilm formation of *Enterococcus faecalis* and *Streptococcus agalactiae*, most likely by inhibiting the production of extracellular polymeric substances. 

The antibiofilm activity of honey against periodontic bacteria embedded in biofilm has not been intensively investigated. Honey inhibits the growth and biofilm formation of *S. mutans* at concentrations between 12.5% and 50% [51]. The exosome-like extracellular vesicles (containing defensin-1) found in honey exhibit pronounced antibiofilm activity against *S. mutans* in comparison to *S. sanguinis* [54]. In another study [64], inhibition of *P. gingivalis* biofilms and a reduction in the number of visible bacteria within 42-hour-old biofilms were observed in the presence of diluted honey at a concentration of 10% (manuka and non-manuka honey). 

## 4. Honey in the Cariogenic Process

It is well known that dietary sugars, particularly sucrose, contribute significantly to the progression of dental caries and the demineralisation process and promote the formation of oral biofilm. The composition of honey and its acidity seem to have a favourable effect on the cariogenic process. In fact, sucrose or its individual monosaccharide units (glucose and fructose) selectively promote acidogenic and acid-tolerating bacterial species, including *S. mutans*. 

Acidic solutions that come into contact with teeth can cause dental erosion. Any solution with a pH value lower than 5.5, a critical value for dental enamel, may cause dental erosion. The acidity of honey is due to the presence of organic acids, particularly gluconic acid. Gluconic acid, accumulating to a concentration of between 8.6 and 60 mM, is the most abundant acid in honey and the major determinant of its acidity (pH 3.4–4.5). The erosive effect of honey has been investigated in two studies [50,65]. Hablutzel et al. (2018) tested three different types of honey, including manuka honey, for their erosive effect. All honeys, even after dilution with saliva, exhibited a pH value below 5.8. Despite the low pH of these honeys, no erosive activity on the enamel surface was detected. This is in agreement with Grobler and co-workers, who showed that honey, despite its low pH, does not cause erosion after 30 min in contact with teeth [65]. 

Demineralisation is the process that plays a first and key role in dental caries development. Demineralisation begins at the atomic level at the crystal surface inside the enamel or dentine and can continue unless halted, with the endpoint being cavitation [66]. In fact, many cycles of demineralisation and remineralisation continue in the mouth as long as there are cariogenic bacteria, fermentable carbohydrates and saliva present. Due to honey’s composition, it is likely that honey may take part in and stimulate the demineralisation process. Interestingly, several studies have reported a low demineralisation effect. Comparison in vitro evaluations of enamel demineralisation depth by five sweeteners (sucrose, fructose, palm sugar, sucralose and honey) clearly showed that artificial (sucralose) and natural (honey) sweeteners have a lower cariogenic potential than sucrose [67]. The results of previous studies have also shown that honey exhibits a lower demineralisation effect compared to fructose and glucose [68], even lower than that of sucrose [69]. On the other hand, natural honey is able to remineralise the enamel surface in vitro, as shown in two recent studies [70,71]. 

There are several candidates regularly found in honey that may inhibit the cariogenic process. Bee-derived enzyme GOX, a regular but quantitively variable glycoprotein of honey [41], may take part in the peroxidase system as a combination of GOX, lactoperoxidase and iodide [72]. Enzymes such as lactoperoxidase and GOX are used as antimicrobial agents in oral care products. The combination of these two enzymes has already been clinically tested in the 1980s, with the results of reducing dental plaque and gingivitis scores [73,74]. Consistent with these investigations, Paque et al. reported that enzyme-containing toothpaste exhibited comparable efficacy in anti-plaque and anti-gingivitis activity compared with formulations based on sodium lauryl sulphate and triclosan [75]. GOX is a natural and attractive ingredient in toothpaste and dentifrices and is used as an active antibacterial ingredient in dental products.

Besides GOX, polyphenols represent a group of biologically active secondary metabolites commonly found in honey. However, the polyphenolic composition is very diverse, depending on the botanical and geographical origins of the honey. Several types of polyphenols have antimicrobial properties and can inhibit bacterial growth, adherence and biofilm formation (reviewed in [76]). Data from clinical studies show a high degree of heterogeneity [77], and it is currently difficult to draw clear conclusions; nevertheless, polyphenols offer a great perspective in dental medicine. 

## 5. Clinical Evidence of Honey in the Prevention of Dental Caries

An important factor in the management of dental caries is the reduction and/or elimination of cariogenic bacteria in both planktonic and biofilm-embedded states. Although the application of systemic antibiotics early in the prevention or treatment of dental caries showed some potential efficacy, their usage has been gradually reduced in recent decades [78]. Antimicrobials such as chlorhexidine, iodine, ozone, quaternary ammonium salts and antimicrobial peptides, as well as natural products (e.g., essential oils), are already used in clinical settings for managing the development of carious lesions [78,79]. 

**Figure 1:**
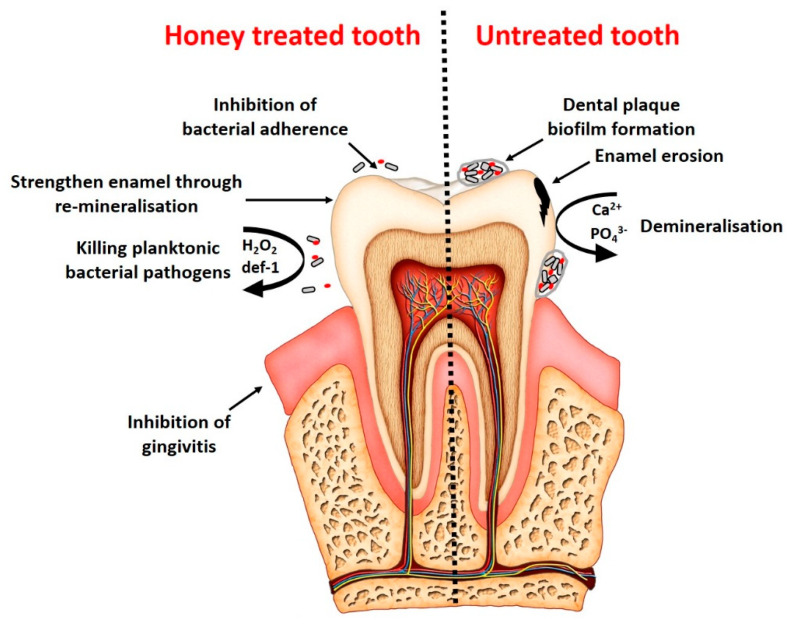
A schematic overview of the most dominant biological effects of honey in the prevention of dental caries.

The promising antibacterial and antibiofilm activity of honey and its constituents and results from a recent systematic review [46] have promoted its use in the management of dental caries (Figure 1). However, the findings from laboratory studies investigating the antibacterial activity of honey may not be directly translated into clinical studies related to dental caries. 

In order to identify clinical studies where honey was used as an antibacterial product, electronic scientific databases PubMed, Scopus and Web of Sciences were used for the literature search by using the following keywords and their combination: “honey”, “dental”, “caries” and “plaque”. Articles published between 2000 and 2022 with full-text availability in English were included in the study.

J.D. and J.M. carried out an independent literature search and selected and assessed publications that were later compared by both authors in order to eliminate duplicate records. Any disagreement was resolved by discussion to reach a consensus.

Overall, nine clinical studies were identified where honey was used as an anti-cariogenic agent (Table 2). A pilot clinical study evaluating the anti-plaque effect of manuka honey was conducted in 2004 [80]. In this study, manuka honey significantly reduced the plaque score after a 21-day trial, but no changes were observed in a control group (sugar-free chewing gum). Similarly, manuka honey was used in a single-blind clinical study, and its anti-plaque efficacy was compared to that of chlorhexidine mouthwash and xylitol chewing gum in 60 subjects [81]. Again, manuka honey was shown to be comparably effective to chlorhexidine in reducing the plaque score and superior to xylitol chewing gum after 3 days of treatment. Lastly, the third clinical controlled trial using manuka honey showed similar efficacy in reducing the plaque score and *S. mutans* count between chlorhexidine and manuka honey after 7 and 14 days of treatment [82].

A double-masked parallel clinical trial based on a 4-day plaque regrowth model was conducted on 66 subjects who were divided into three groups of 22, each using a different mouthwash (chlorhexidine, honey and saline). Honey, although less potent than chlorhexidine, was effective against putative periodontal pathogens and significantly reduced plaque formation [83]. The aim of another randomised controlled clinical trial was to investigate the effect of honey on plaque formation and on dental plaque bacterial counts in 20 subjects divided into three groups (honey, sucrose and sorbitol) [84]. Significant differences in plaque pH values were observed in the honey and sucrose groups compared to those observed in the sorbitol group. Chewing of pure honey for 2 min resulted in a drop of pH value but not below the critical decalcification pH of 5.5. In contrast, rinsing with 10% sucrose solution for 1 min caused a decrease in pH value to below 5.5. In addition, bacterial counts were significantly reduced only in the honey group compared to the other treatment groups. The dental plaque score in 56 subjects who used a 5% honey solution as a mouthwash for a period of 6 days was determined by Alibasyah et al. (2018) [86]. A significant difference between dental plaque score before and after 6 days of using honey solution was found. 

Honey is often used as a mouthwash in the clinical testing of the treatment of periodontal diseases [89]. The effectiveness of three types of mouthwash (manuka honey, raw honey and chlorhexidine) on plaque scores was evaluated in a double-blind, randomised, controlled field clinical trial [87]. A total of 124 children were divided into three therapeutic groups: manuka honey—40% solution (n = 41), raw honey—20% solution (n = 41) and chlorhexidine—0.2% solution (n = 42). Each mouthwash (10 mL) was used twice daily for 21 days. Plaque scores were examined at baseline, on the 22nd day (1 day after mouthwash discontinuation) and on the 28th day (1 week after mouthwash discontinuation). Among the three types of mouthwashes, chlorhexidine was found to be the most effective in reducing plaque. However, both honey-type solutions also significantly reduced plaque formation and were demonstrated to have equal effectiveness. 

The antibacterial efficacy of chlorhexidine, honey and propolis against an *S. mutans* load was clinically evaluated in 60 children [88]. Although all three antibacterial products were effective in reducing *S. mutans* loads (over 76% reduction) after 30 s of mouth rinsing, the authors of the study did not include a control group (e.g., saline solution) in the clinical study. It is likely that mouth rinsing with plain water or mild saline solution does not change the bacterial load of *S. mutans,* as proved clinically elsewhere [90], although different clinical conditions were applied.

Different exclusion criteria were applied in clinical studies listed in Table 2. Participants with a history of systematic diseases/conditions were excluded from most of the clinical studies. Interestingly, the history of allergy to honey was not considered an exclusion criterion in all studies.

## 6. Honey Quality Matters

Honey intended for human consumption must meet precisely defined composition criteria, including the sugar, moisture and water-insoluble solids contents, electrical conductivity, free acids, diastase activity and hydroxymethylfurfural content. Honey quality criteria are based on international honey standards, which are specified in the European Honey Directive (2002) and in the Codex Alimentarius Standard for Honey (2001) [17]. 

Currently, none of the legislative criteria includes information about the biological activity of honey, including antibacterial activity, despite the widely accepted fact that every type of honey exhibits an antibacterial effect. The honey samples used for pre-clinical and clinical testing, either topical or systemic application, need to be characterised in detail together with their biological properties. Honey that fulfils all current legislative parameters but is exposed to high temperature or prolonged storage may exhibit very weak antibacterial activity equal to the activity of the sugar content only. This issue has already been discussed elsewhere [21]. 

Another issue faced by researchers and clinicians is the sterility of honey recommended for clinical testing. None of the above-mentioned clinical studies tested honey that had been sterilised or microbially filtered, which is usually carried out by gamma irradiation. In the case of topical application, honey sterilisation is obligatory in wound care management. Several studies have reported that gamma radiation of honey has been shown to eliminate vegetative microbial cells as well as microbial spores [91,92,93] without affecting the overall antibacterial activity of the honey [91,93,94,95]. Although H_2_O_2_ levels are not elevated in irradiated honey compared to non-irradiated honey, the concentration of defensin-1 is significantly reduced in irradiated honey [96]. Finally, the antibiofilm activity of irradiated honey is not negatively affected as it effectively reduces established biofilms of *S. aureus* and *P. aeruginosa* [96].

One of the biases in clinical trials using honey to prevent dental plaque is insufficient information about the tested honey and in vitro antibacterial efficacy. In order to perform a comparative analysis of clinical studies, it is necessary to adopt a universal method for the antibacterial activity testing of honey. The broth microdilution method is the most suitable and appropriate method for evaluating the activity of honey [97]. 

## 7. Conclusions 

From a clinical point of view, honey is an attractive and effective therapeutic agent recommended primarily for topical application in diverse clinical disciplines, including dermatology, ophthalmology and stomatology. The most beneficial property of honey is antibacterial/antibiofilm activity, which also represents its most studied biological effect. In fact, its antibacterial in vitro efficacy against a broad spectrum of wounds and periodontal pathogens has clearly been proved. Although honey has been shown to reduce the bacterial content of dental plaque, its clinical efficacy is less potent than that of conventional mouthwash containing chlorhexidine. One of the major drawbacks of most clinical studies is the quality of honey and the absence of laboratory testing of the honey used. Therefore, the quality of honey and its antibacterial and antibiofilm activity need to be determined before pre-clinical and clinical trials are conducted. The antibacterial, and particularly antibiofilm, effect of honey is an important factor in determining honey’s clinical efficacy in the prevention of dental caries through the eradication of viable bacteria within dental plaque biofilm. 

We argue that the information presented in this perspective review will stimulate further clinical research on honey in periodontal diseases where its antibacterial/antibiofilm as well as anti-inflammatory and wound healing activities will be employed. 

## Figures and Tables

**Table 1:** Dominant antibacterial constituents of honey and their mechanisms of action against oral bacteria.

Antibacterial Factor/Compound	Origin	Target Bacteria		Mechanism of Action	Ref.
		Gram-Positive	Gram-Negative		
MRJP1	bee	+ *	+ *	Disruption of cell wall integrity *	[32,33,34]
defensin-1	bee	+	+	Decreased bacterial cell hydrophobicity and disruption of cell membrane permeability	[35,36,37,38]
H_2_O_2_	bee	+	+	Destruction of cell wall integrity. Lipid peroxidation and damage to bacterial cell proteins and DNA	[39,40,41]
gluconic acid	bee	-	+	Membrane depolarisation and destruction	[42]
MGO	plant	+	+	Oxidative stress by reacting with cellular proteins and DNA	[43,44,45]

* Reported antibacterial activity remains controversial.

**Table 2:** Summary of data collected from human clinical studies using honey as an anti-plaque agent.

Type of Honey	Honey Concentration	Honey Sterility	Control	Participants	Outcomes	Year	Ref.
MH UMF 15+	100%	No	0.2% CHX Sugar-free chewing gum	MH group (n = 15) Sugar-free chewing gum (n = 15)	In a pilot clinical study, MH was able to significantly reduce the plaque score after a 21-day trial period. On the other hand, no significant changes were observed in the control group.	2004	[80]
MH	100%	No	0.2% CHX Xylitol chewing gum	MH group (n = 20) CHX group (n = 20) Xylitol chewing gum group (n = 20)	In a single-blind study, MH and chlorhexidine mouthwash significantly reduced plaque formation in comparison to xylitol chewing gum after 3 days of use.	2010	[81]
Multifloral	50%	No	0.2% CHX Saline	Honey group (n = 22) CHX group (n = 22) Saline group (n = 22)	In a double-masked parallel clinical trial based on a 4-day plaque regrowth model, honey, although less potent than chlorhexidine, reduced plaque formation.	2012	[83]
Unspecified honey	100%	No	10% sucrose 10% sorbitol	n = 20 *	Significant differences in plaque pH were shown between the honey and sucrose groups compared to the sorbitol group. Among the mouthwashes tested, only honey was able to significantly reduce the number of bacteria that were recovered from plaques 30 min after exposure.	2014	[84]
Unspecified honey	100%	No	0.2% CHX Xylitol	Honey group (n = 30) CHX group (n = 30) CHX + xylitol group (n = 30)	In a single-blind randomised control trial, all groups were effective in reducing plaque. The honey group was more effective than the CHX group but comparable with the CHX + xylitol group over periods of 15 and 30 days.	2015	[85]
Tongra honey	5%	No	None-	Honey group (n = 54)	A significant difference was shown between dental plaque scores before and after using honey as a mouthwash for a period of 6 days.	2018	[86]
MH RH	40% (MH) 20% (RH)	No	0.2% CHX	MH group (n = 45) RH group (n = 45) CHX group (n = 45)	All tested mouthwashes showed significant reductions in plaque scores. CHX was most effective in reducing plaque. No differences in efficacy were documented between MH and RH.	2018	[87]
Unspecified honey	50%	No	0.12% CHX 5% propolis	Honey group (n=20) CHX group (n = 20) Propolis group (n = 20)	All tested mouthwashes showed an immediate and direct reduction of *S. mutans* load. CHX was the most effective, followed by propolis and honey.	2021	[88]
MH UMF 15+	100%	No	0.2% CHX	MH group (n = 30) CHX group (n = 30)	In a randomised controlled trial, no significant differences in plaque score and *S. mutans* count were found between groups after 7 and 14 days of treatment.	2021	[82]

MH, manuka honey; RH, raw honey; CHX, chlorhexidine gluconate; UMF, unique manuka factor. * Number of subjects in therapeutic groups was not shown.

## Data Availability

Not applicable.
